# Concurrent Hemolytic Anemia and Methemoglobinemia Revealing Glucose-6-Phosphate Dehydrogenase (G6PD) Deficiency in an Infant Following Peanut Ingestion

**DOI:** 10.7759/cureus.105458

**Published:** 2026-03-18

**Authors:** Nada Ben Mechlia, Hafsa A Redouane, Maryam O Al-Tamo, Laian Hamod, Rufaida Mohamed

**Affiliations:** 1 Pediatric Emergency Medicine, Al Jalila Children's Speciality Hospital, Dubai, ARE; 2 Medicine, Dubai Medical University, Dubai, ARE; 3 General Practice, Dubai Medical University, Dubai, ARE; 4 Medicine and Surgery, Dubai Medical University, Dubai, ARE; 5 Internal Medicine/Surgery, Dubai Medical University, Dubai, ARE

**Keywords:** cyanosis, food-induced hemolysis, g6pd deficiency, methemoglobinemia, oxidative stress

## Abstract

Glucose-6-phosphate dehydrogenase (G6PD) deficiency is among the most common enzyme disorders affecting red blood cells. It often remains unnoticed until the individual is exposed to oxidative stress, which can trigger rapid hemolysis. Methemoglobinemia develops when hemoglobin becomes oxidized into a form that cannot efficiently transport oxygen. This leads to impaired oxygen delivery to tissues and cyanosis that does not improve despite supplemental oxygen. Although uncommon, methemoglobinemia can develop as a serious complication during episodes of severe oxidative stress. We describe the case of an 11-month-old boy who developed acute hemolysis together with methemoglobinemia shortly after his first exposure to peanuts.

## Introduction

Glucose-6-phosphate dehydrogenase (G6PD) deficiency is a frequently encountered inherited enzyme disorder affecting red blood cells. In many patients, the condition does not cause symptoms initially and may remain undiagnosed until an episode of oxidative stress occurs. Such exposure can trigger acute hemolysis, most commonly due to infections, medications, or certain foods [1]. Methemoglobinemia is a condition in which hemoglobin is oxidized, resulting in reduced oxygen delivery to tissues. Acute hemolytic anemia and methemoglobinemia may occur simultaneously; this process is associated with depletion of NADPH and glutathione, leading to marked oxidative injury, hemolysis, and the conversion of hemoglobin to methemoglobin. Clinically, it presents with cyanosis and low oxygen saturation that does not significantly improve with supplemental oxygen. Although methemoglobinemia is well described in older children and adults, it is relatively uncommon in infants and may not be recognized early in the differential diagnosis [2].

In patients with G6PD deficiency, oxidative stress can occasionally lead to both hemolysis and methemoglobinemia occurring simultaneously. This association is clinically important, as the standard treatment for methemoglobinemia, such as methylene blue, may worsen hemolysis in these patients [3]. We report the case of an 11-month-old infant who developed acute hemolysis with concurrent methemoglobinemia shortly after he was fed peanuts in crushed form, highlighting a rare trigger and presentation of G6PD deficiency in early infancy.

## Case presentation

An 11-month-old previously healthy male infant, from the Middle East, with an unremarkable family history, was brought to the emergency department with a sudden onset of pallor and central cyanosis shortly after ingesting peanuts for the first time. There was no history of choking, rash, wheezing, vomiting, or fever. The child remained alert and interactive. Table 1 shows the patient's vital signs at presentation.

**Table 1:** Vital signs of the patient at presentation

Vital sign	Value	Reference range
Temperature	37 °C	36.5–37.5 °C
Heart rate	130 beats/min	80–160 beats/min
Respiratory rate	30 breaths/min	25–40 breaths/min
Oxygen saturation	84% (room air)	≥95%

The laboratory findings revealed severe anemia with reticulocytosis, elevated lactate dehydrogenase (LDH), and low haptoglobin, consistent with acute hemolysis (Table 2). The direct Coombs test was negative, arguing against autoimmune hemolytic anemia. The elevated methemoglobin level indicated concurrent methemoglobinemia.

**Table 2:** Blood test results of the patient at presentation G6PD: glucose-6-phosphate dehydrogenase

Parameter	Result	Reference range
Hemoglobin	7.7 g/dL	10.5–13.5 g/dL
Reticulocyte count	Elevated	0.5–2.5%
Lactate dehydrogenase (LDH)	500 IU/L	140–280 IU/L
Haptoglobin	<0.1 g/L	0.3–2.0 g/L
Direct Coombs Test	Negative	Negative
Methemoglobin	7.10%	<1.5%
Chest Radiography	Normal	Normal
G6PD Screening	Pending	—

High-flow nasal cannula oxygen therapy was initiated at 2 L/kg/min with 100% inspired oxygen, but there was minimal improvement in oxygen saturation. Given the severity of anemia and hypoxia, a packed red blood cell transfusion was administered. Following the transfusion, the patient’s oxygen saturation and clinical status improved gradually. Because the patient’s G6PD status had not been confirmed, methylene blue was not initiated. The G6PD screening test, performed later at another hospital, was found to be deficient, confirming the diagnosis.

The patient was monitored in the hospital, weaned off oxygen, and discharged home in stable condition after three days. The methemoglobin level had decreased and had returned to normal before discharge. The family was counseled regarding G6PD deficiency, the avoidance of known triggers, and the importance of follow-up.

## Discussion

This report describes an atypical initial presentation of G6PD deficiency in an 11-month-old infant. Following his first exposure to peanuts, he developed sudden acute hemolysis and methemoglobinemia. While hemolytic anemia is a common feature of G6PD deficiency, central cyanosis is rare, particularly in infants [4]. In individuals with G6PD deficiency, oxidative stress can damage hemoglobin within red blood cells and make their membranes fragile, leading to premature cell lysis [1,5]. In our patient, laboratory tests revealed severe anemia, elevated LDH levels, absent haptoglobin, an increased reticulocyte count, and a negative direct Coombs test. Overall, these results suggest acute non-immune hemolysis.

The persistence of cyanosis despite high-flow oxygen therapy was an important diagnostic finding. Unlike hypoxia due to cardiac or pulmonary causes, methemoglobinemia occurs when the iron in hemoglobin is oxidized from the ferrous (Fe²⁺) state to the ferric (Fe³⁺) state [2]. This results in the formation of methemoglobin, which is ineffective at carrying oxygen [2]. In addition, it causes a leftward shift of the oxygen-hemoglobin dissociation curve, reducing oxygen release to the tissues even when arterial oxygen levels are normal [2]. This explains why oxygen supplementation did not significantly improve the patient’s oxygen saturation.

The coexistence of hemolysis and methemoglobinemia complicates treatment. Methylene blue is typically the first-line treatment for severe methemoglobinemia, but it requires NADPH for efficacy. In patients with G6PD deficiency, it may not be effective and can even increase oxidative stress, which could worsen hemolysis [2,3]. It acts as an artificial electron acceptor and requires NADPH as a cofactor to be reduced to leukomethylene blue, which in turn reduces methemoglobin back to hemoglobin. Therefore, supportive care is the main treatment approach. In our case, transfusion of packed red blood cells improved his clinical condition and laboratory findings, underscoring the importance of early initiation of supportive treatment.

Fava beans are the classic trigger for hemolytic crises in patients with G6PD deficiency. However, hemolysis can also occur due to other oxidative stressors, such as infections, certain medications, and various foods [1,5]. Peanut ingestion is not commonly reported as a cause, which makes this case particularly unusual. This suggests that even in the absence of typical triggers, G6PD deficiency should still be considered, particularly in populations with higher prevalence [5]. In infants, persistent cyanosis can be caused by a range of conditions, including congenital heart defects, respiratory illnesses, infections such as sepsis, or disorders of hemoglobin [2]. When a baby remains cyanotic despite receiving oxygen and when cardiac and pulmonary evaluations are normal, it is important to consider a hematologic cause. By identifying the cause early, clinicians can avoid unnecessary investigations and treatments and ensure that appropriate supportive care is provided promptly.

This report presents a rare yet important manifestation of G6PD deficiency in an infant, with concurrent hemolysis and methemoglobinemia. It highlights the need for clinicians to maintain a high index of suspicion when an infant presents with unexplained anemia and cyanosis that does not improve with oxygen therapy, even if the usual oxidative triggers are not present. Recognizing the condition early is important for accurate diagnosis and the initiation of appropriate and safe treatment.

## Conclusions

G6PD deficiency in infancy can present with atypical manifestations involving acute hemolysis accompanied by methemoglobinemia, resulting in cyanosis and hypoxia that are unresponsive to supplemental oxygen. The coexistence of hemolysis and methemoglobinemia poses a therapeutic challenge, as the standard treatment for methemoglobinemia, methylene blue, may exacerbate hemolysis in G6PD-deficient patients. This highlights the importance of early recognition to avoid the introduction of contraindicated therapy and to initiate appropriate supportive management. Other triggering factors, such as dietary exposure, may be potential sources of oxidative stress and hemolysis in G6PD deficiency, unlike the classical triggers, including fava beans. Therefore, clinicians should maintain a high index of suspicion for G6PD deficiency in infants presenting with unexplained hemolytic anemia and cyanosis, even in the absence of a known family history or typical precipitating factors. Overall, this case report underscores the importance of timely diagnosis, careful treatment selection, and family education on trigger avoidance and the potential for future recurrence.
